# Lexical neutrality in environmental health research: Reflections on the term walkability

**DOI:** 10.1186/s12889-017-4943-y

**Published:** 2017-12-08

**Authors:** Samantha Hajna, Nancy A. Ross, Simon J. Griffin, Kaberi Dasgupta

**Affiliations:** 1 MRC Epidemiology Unit, Institue of Metabolic Science, University of Cambridge, School of Clinical Medicine, Box 285, Cambridge Biomedical Campus, Cambridge, Cambridgeshire, CB2 0QQ UK; 2 Department of Geography, McGill University, Montreal, Quebec, Canada; 3 Primary Care Unit, Institute of Public Health, University of Cambridge, School of Clinical Medicine, Cambridge Biomedical Campus, Cambridge, UK; 4 Department of Medicine, McGill University, Montreal, Quebec, Canada; 5 Centre for Outcomes Research and Evaluation, Research Institute of the McGill University Health Centre, Montreal, Quebec, Canada

**Keywords:** Neighbourhood environments, Neighbourhood walkability, Health, Physical activity, Neighbourhood physical activity environments

## Abstract

Neighbourhood environments have important implications for human health. In this piece, we reflect on the environments and health literature and argue that precise use of language is critical for acknowledging the complex and multifaceted influence that neighbourhood environments may have on physical activity and physical activity-related outcomes. Specifically, we argue that the term “neighbourhood walkability”, commonly used in the neighbourhoods and health literature, constrains recognition of the breadth of influence that neighbourhood environments might have on a variety of physical activity behaviours. The term draws attention to a single type of physical activity and implies that a universal association exists when in fact the literature is quite mixed. To maintain neutrality in this area of research, we suggest that researchers adopt the term “neighbourhood physical activity environments” for collective measures of neighbourhood attributes that they wish to study in relation to physical activity behaviours or physical activity-related health outcomes.

## Background

The improper use of language was identified in the *New England Journal of Medicine* in 1846 as an important source of medical error: “*[physicians] not [un]frequently make use of language which is unintelligible to most persons; and whether their own views are correct or incorrect, the consequence is, that people are led in error (p.g., 378)*” [1] For example “states of the pulse” was a term central to medical diagnoses during the nineteenth century and could refer to a pulse or to blood that was either “hard”, “soft”, “wiry”, “corded”, or “suffocated”. Each of these figurative terms would be difficult to interpret and often led to dubious treatments by physicians [1]. As medicine progressed, the term “states of the pulse” was replaced with more precise terms that quantified the underlying ‘components’ of blood (e.g., pulse rate and blood pressure). This ultimately reflected a better understanding of pathophysiological mechanisms and improvements in treatment. Herein, we reflect on the environments and health literature and argue that, similar to “states of the pulse”, the term “neighbourhood walkability” is misleading because the term shifts our focus away from other potentially important outcomes and inherently infers that a universal causal association exists between neighbourhood factors and walking despite a heterogeneous evidence-base.

## Main text

Researchers seek answers to questions by weighing the evidence and drawing conclusions based on what the data support - not based on what they hope or believe should be observed. Unfortunately, this is not what occurs in many clinical and public health studies. A reliance on statistical significance rather than on the interpretation of variance estimates in the context of clinical importance, has led many researchers to draw incorrect conclusions [2]. This is further compounded by the fact that positive effects are more likely to be reported and published than small or null effects [2, 3]. We caution environmental health researchers not to fall into the same, albeit more subtle, trap of propagating a biased message through the use of non-neutral language. Specifically, in the context of the environmental health literature, we caution researchers about the term “neighbourhood walkability”.

The term “neighbourhood walkability” was introduced into the lexicon of the transportation and public health literatures in the early 2000’s - a time when researchers began to suspect that neighbourhood designs might play an important role in either promoting or restricting physical activity. It was and continues to be used today to describe the features of neighbourhoods that are collectively thought to promote physical activity or to be associated with physical activity-related health outcomes. The first problem with the term “neighbourhood walkability” is that it conflates the exposure name with the name of one specific behavioural outcome (i.e., walking), thereby inherently identifying this single outcome as important and downplaying the potential influence of neighbourhoods on other physical activity outcomes (moderate-to-vigorous intensity physical activity (MVPA) in the forms of jogging, running, and cycling, for example).

To see the limitations of the term “neighbourhood walkability” more clearly, it is useful to consider the terms used in other environmental health research domains. For example, researchers investigating the links between air pollution and asthma might label their global exposure measure as “the air quality index”. These authors would not, however, use the term “neighbourhood breathability” – since air quality may be linked to a variety of respiratory-related outcomes – not just breathing. This is essentially what we are doing when we use the term “neighbourhood walkability”. We are encouraging a narrow understanding of the role of neighbourhoods on human health by linking it just to walking. To maintain neutrality in our research and to ensure that one outcome is not given more emphasis than another, we advocate for the use of a term that does not conflate the name of the exposure with that of a single outcome.

We are not the first group to reflect on the term “walkability” and the implications that this might have for the direction of science [4, 5]. For example, Ann Forsyth shed light on the complexity of the term, both in terms of how it is defined differently in different contexts and the problems that these different definitions might have on translating research into action [4]. Lise Gauvin and colleagues also raised concerns with the term “walkability” - identifying “walkability” as a misnomer since environmental factors may be linked to other forms of physical activity – not just walking [6]. They also raised concerns regarding the term’s lack of consideration of human agency and the diverse set of environmental contexts and factors that may be important in facilitating active lifestyles. The authors suggested replacing the term “walkability” with “neighbourhood active living potential” - a term that would better acknowledge individuals’ capacities to choose a particular course of action (i.e., potential) and that would capture all of the social and physical neighbourhood features that might predict one’s probability of being physically active [6]. We believe that Lise Gauvins' and colleagues' concerns regarding the term “walkability” are important as they have the potential to influence our thinking regarding the role of environments in human health. Despite their important work in this area, there remains a heavy reliance on the term “walkability” in the literature today. In our piece, we reiterate the concerns raised by others relating to the term “walkability” and build upon their work by identifying another reason why researchers may want to consider avoiding using the term “walkability”.

In addition to the concern that “walkability” is linked to one single outcome, the term inherently implies that a universal causal association exists between the factors that comprise summary measures of neighbourhood features and walking. While “neighbourhood walkability” has indeed been linked to walking in some studies [7], the associations have been small or non-existent in others [8, 9] - varying according to factors such as outcome measurement [9], and geographic and social context [10]. For example, positive associations have been observed in Europe, China, and Japan when both walking and walkability have been objectively-assessed using physical activity monitors and Geographic Information Systems measures or direct neighbourhood audit, but the evidence-based is less clear in North America [10]. By presuming a universal association exits, the complexities of this relationship in different settings and contexts are ignored, as are the implications that these may have on informing public health policy.

Many articles have been published in the last decade on the role of “neighbourhood walkability” on human health behaviours and health outcomes. The term has become central to the public health lexicon. It is perhaps not difficult to understand its popularity given that the public health community in the United States has asked the question “*Can walking be used as a unifying theme for other realms of public health such as physical activity, safety, air pollution and social capital? (p.g., 1503)”* [11] Replacing the term “neighbourhood walkability” with a more neutral term may be challenging as it has become an ingrained part of our lexicon. Still, we believe it necessary in order to avoid falsely inferring that a universal association exists despite evidence to the contrary or downplaying, just by virtue of our language, the variety of physical activity outcomes to which features of neighbourhood may be linked.

There are several alternatives to the term “neighbourhood walkability”. First, researchers could use terms that are specific to the exposures of interest (e.g., green spaces) [4]. Such nomenclature allows for the possibility that the associations between these exposures and health outcomes (e.g., incident diabetes) may not necessarily be mediated through physical activity alone but also through other factors, such as food environments. Alternatively, for researchers who like the collective nature of the term “neighbourhood walkability” we recommend that they use a term that encompasses the construct that they intend to capture without implying that a universal causal association exists with one particular outcome. One such example is “neighbourhood physical activity environments”. This term is sufficiently broad enough to not elevate the importance of a single type of physical activity over others, yet specific enough to acknowledge the physical activity potential of neighbourhood characteristics which will help to distinguish it from global measures of neighbourhood environments that are intended to capture different constructs (e.g., the “food environment” for the assessment of diet-related behaviours and outcomes). If the focus of the collective terms of neighbourhood environments are both social and physical as described by Lise Gauvin and colleagues (i.e., consisting of the following three underlying dimensions: activity friendliness, safety, and density of destinations) [6], their proposed alternative “neighbourhood active living potential” is also a good option because it neither links itself to one outcome nor implies causality – thereby remaining neutral to the associations under investigation. For researchers requiring a collective term that does not encompass social factors – the term “neighbourhood physical activity environments” may be preferred.

When proposing any change in lexicon, it is important to consider the implications that this change could have not only on the direction of research, but also on the message that is being conveyed to the public. The advantages of the term “walkability” are that it is catchy, simple, and easy to understand. There is no doubt that walking does indeed matter for health - especially for older adults [12]. “Walkability” is not far off the message that public health advocates want to convey but implies that neighbourhood environments are universally associated with walking despite mixed evidence in some contexts. This may lead to the development of interventions that are either inappropriate in certain populations and contexts or that do not recognise the complex effects that they might have on multiple physical activity-related behaviours and outcomes. Replacing the term “neighbourhood walkability” with “neighbourhood physical activity environments” would help us send a clearer public health message - a message that both acknowledges: 1) the possibility of important variability in findings across different contexts, study populations, and methodologies; and 2) the breadth of influence that neighbourhoods may have on different types of physical activity – not just walking.

## Conclusions

In the words of Oli Miettieten, we need to be *“independent agents rather than merely perpetuators of the past (p.g., 498)”* [13] and we would argue that this includes reconsidering our use of language however embedded in our lexicon it may be. If not, we run the risk of repeating mistakes, letting preconceptions guide our science, and increasing research without increasing knowledge [13]. We are among the many researchers who have used the term “neighbourhood walkability” in our research. We believe, however, that a shift in lexicon is important for acknowledging the multifaceted influence that neighbourhood environments may have on physical activity behaviours and ultimately on human health. To this end, we encourage researchers to adopt a term such as “neighbourhood physical activity environments” in relation to studies examining the link between neighbourhood environments and physical activity behaviours and physical activity-related health outcomes.
